# The sequelae of septic hip arthritis in children: a revised classification and case review

**DOI:** 10.1080/07853890.2025.2553878

**Published:** 2025-09-05

**Authors:** Bohai Qi, Qingda Lu, Xiaowei Wang, Qiang Jie, Fei Su, Chenxin Liu, Yating Yang

**Affiliations:** Pediatric Orthopaedic Hospital, Honghui Hospital of Xi’an Jiaotong University, Xi’an, Shaanxi, China

**Keywords:** Children, classification, septic arthritis of hip, sequelae

## Abstract

**Background:**

Existing classification systems for sequelae of pediatric septic arthritis of the hip (SAH) are notably complex. This study introduces a simplified radiographic classification—the Xi’an Honghui Hospital Paediatric Orthopaedic Classification (HHPO classification)—designed to enhance accuracy in treatment planning and prognostic evaluation.

**Methods:**

A retrospective analysis was conducted involving 18 pediatric patients with SAH. Pelvic radiographs were evaluated to assess the structural integrity of the femoral head and neck and their spatial relationship with the acetabulum. Based on these assessments, the HHPO classification was developed. Six independent observers classified each case using both the HHPO and Choi systems. Interobserver reliability and two-week intraobserver consistency were assessed and compared between the two classifications.

**Results:**

The distribution according to the HHPO classification was as follows: Type IA (*n*=5), Type IB (n=6), Type ID (*n*=2), Type IIA (*n*=3), and Type IIB (*n*=2). The HHPO system demonstrated significantly higher inter- and intraobserver agreement compared to the Choi classification. Clinically, severe hip pain was reported in 5 patients, occasional pain in 2, and no pain in 11. The majority of hips (88.9%) exhibited satisfactory range of motion, and 72.2% were radiologically stable. Earlier onset of infection was associated with more severe sequelae.

**Conclusion:**

The HHPO classification is simpler, more reproducible, and demonstrates potential clinical utility for managing pediatric SAH sequelae in this cohort.

## Introduction

During the course of septic arthritis of the hip (SAH) in children, bacterial toxins produced from breakdown products produced by white blood cells can damage the anatomical structures of the hip joint, including but not limited to the articular cartilage, the acetabulum, the proximal femoral metaphysis, and the growth plate [1,2]. Delayed diagnosis and treatment can exacerbate the damage, potentially resulting in restriction of hip joint function, pain, a leg length discrepancy, and limping, and can lead to early onset osteoarthritis [3–8]. The early diagnosis of other pyogenic arthritis, including hip joint, is based on the patient’s complete medical history, physical examination, imaging studies, laboratory tests, and suspected joint aspiration [9]. Kocher et al. [10,11] proposed four indicators for predicting SAH: (1) the affected limb refuses to bear weight or limps; (2) fever with a maximum body temperature >38.5 °C; (3) white blood cell count >12 × 10^9^/L; (4) erythrocyte sedimentation rate (ESR) >40 mm/h. If a child meets all four diagnostic criteria, the accuracy of diagnosing SAH is 93%; Caird et al. [12] added a fifth predictive indicator to Kocher’s four diagnostic criteria: CRP >20 mg/L. In terms of treatment, Caldaci et al. [13] proposed that the treatment plan for children with pyogenic arthritis is if there is no improvement in the condition within 24 h of empirical antibiotic use, surgical intervention is required; the choice among joint aspiration, arthrotomy, and arthroscopy depends on the surgeon’s skill and the time elapsed since the onset of symptoms.

The surgical management of sequelae in SAH strives to reconstruct the structure and biomechanics of the hip joint, enhance hip function, and establish a basis for preserving optimal hip function throughout adulthood. To guide surgical programming and prognostic assessment, there are several relevant classification systems. Of all the classification systems, the one proposed by Choi et al. [14] is the most commonly reported in the literature and is similar to that of Hunka et al. [15]. The Choi classification system categorizes the degree of erosion in the femoral head and neck resulting from bacterial toxins during the inflammatory response. It is initially classified into four main types, which are further divided into eight subtypes based on various secondary deformities of the femoral head and neck. However, as cartilage structures in the hip joint may not be well-visualised on X-rays in young children, applying this classification system may not provide utterly realistic information, and it is cumbersome to apply and difficult to remember. Although previous studies [16,17] have made various modifications to the Choi classification, we believe that the relationship between the femoral head and the acetabulum (stability) and the degree of disruption in the femoral head-neck structure are the two most important aspects when assessing treatment and prognosis. Therefore, our research aims to integrate previous literature reports and our own practical experience to propose a modified classification– the Honghui Pediatric Orthopaedic Modified Classification(HHPO), hoping to provide convenience for physicians in the classification of SAH.

## Materials and methods

### General information

To review cases of sequelae after acute phase treatment of SAH between October 2014 and January 2023 in our institution. Some patients had received treatment at other medical institutions during the acute phase and were subsequently referred to our institution due to complications or residual deformities. These referrals were issued *via* an online consultation to access patient medical records from their treatment at an external institution. All the included cases exhibited a typical history of SAH and presentation of residual deformity. The diagnosis was confirmed without the need for additional bacterial culture. We collected pelvic X-rays from the early stages of all cases, concealed patient-related information, and distributed the PPT to six pediatric orthopedic doctors, including two senior physicians, two attending physicians, and two resident physicians. Before viewing the PPT, we explained the principles of the HHPO classification and the Choi classification to them, and asked them to independently assess and classify each case. We did not specify the duration for evaluating each case, but required them to re-evaluate each case two weeks later. Finally, we collected and analyzed all the results. The study was approved by the ethical committee of the author’s institution (approval number: 202308004). Written informed consent to publish identifying details (including but not limited to photographs, clinical details, and unique identifiers) was obtained from all participants or their legal guardians. All efforts were made to anonymize participants to the greatest extent possible. The consent to publish is separate from the consent to participate in the study, and written informed consent was provided.

Inclusion criteria: 1. Age ≤14 years, with a history of SAH, and the presence of residual hip deformity; 2. The evaluation of the efficacy of reconstructive surgery must be conducted with a follow-up of at least 2 years post-surgery; 3. Treatment of residual deformities after SAH was conducted in our hospital. Exclusion criteria include individuals without any residual deformity (equivalent to type IA in the Choi et al. classification without residual deformity), tuberculous, and neurogenic.

### Patients

Eighteen children in all participated in this study. There were eight female patients and ten male patients. The condition first manifested between the ages of 20 days and 13 years. Nine patients received immediate treatment in our hospital; nine others were treated at a hospital outside of our institution. With eight patients on the right side and ten on the left, unilateral involvement was seen in all cases. Four patients had methicillin-sensitive Staphylococcus aureus(MSSA), five had methicillin-resistant *Staphylococcus aureus* (MRSA), one had *Citrobacter youngae*, and eight additional patients had unidentified causal organisms. Fifteen patients got open hip drainage, 2 underwent closed puncture drainage, and 1 underwent no surgery during the acute phase. Ten patients received intravenous vancomycin, five received cephalosporin antibiotics, three received oral combination antibiotics, and three received no antibiotic treatment. Reconstructive surgery was performed in 5 patients, with four undergoing pelvic osteotomy combined with proximal femoral osteotomy and one undergoing Greater trochanteric advancement (GTA) combined with limb lengthening. Postoperative imaging showed stable hip joints and better hip function in these five patients. Non-operative cases (IA/IB subtypes) had substantially shorter follow-up (median 3.2 years, range 2–4 years) versus reconstruction cases (minimum 5.5 years). 9/18 cases (50%) were tertiary referrals for complex reconstruction, potentially introducing selection bias toward severe manifestations.

### Radiological classification

This investigation examines a group of patients with residual deformity from SAH and refines the Choi classification to make it intuitive, easy to remember and understand (see Table 1). The following specific subtypes create for the revised classification: Type I: undislocated hip (stable), including IA: valgus or varus deformity of the hip, IB: abnormal femoral head, IC: pseudoarthrosis formation, and ID: femoral head absence (including incomplete absence); and Type II: acetabular dysplasia or dislocation of the hip (unstable), including IA: presence of the femoral head, and IIB: femoral head absence.

**Table 1.** Sequelae of septic arthritis of the hip in children-HHPO classification.

Type	Subtypes	Schema
Type I: Undislocated hip (stable)	IA: Hip valgus or varus	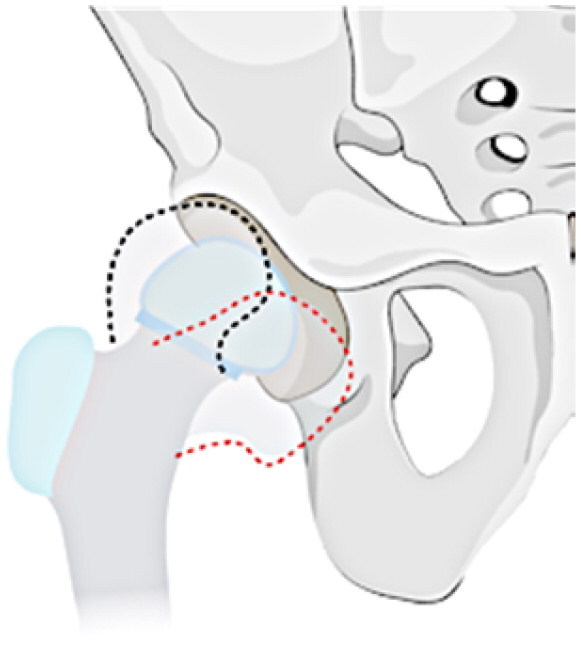
	IB: abnormal femoral head	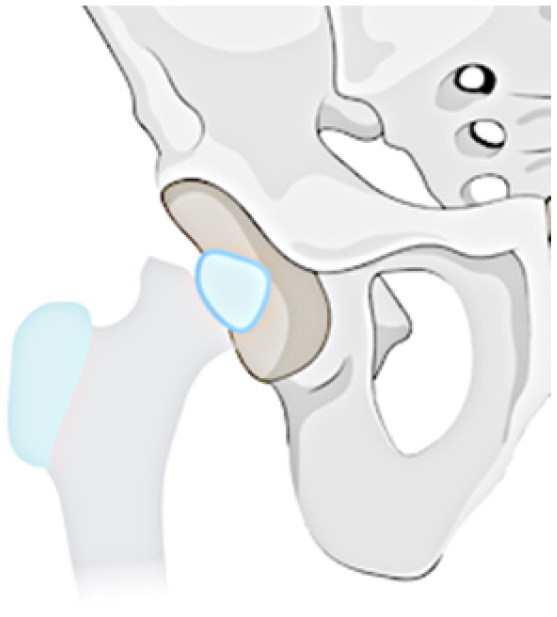
	IC: pseudarthrosis	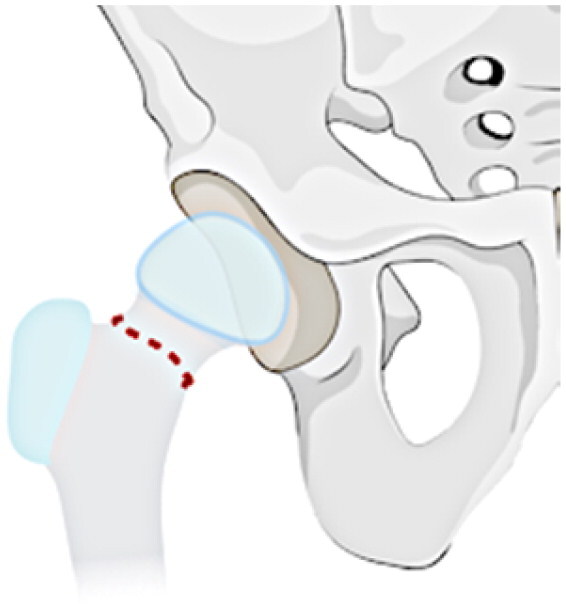
	ID: femoral head absence (including incomplete absence)	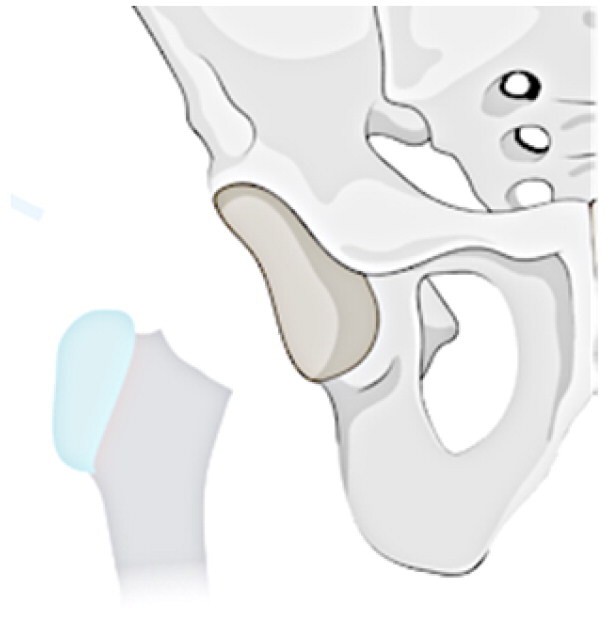
Type II: Acetabular dysplasia or hip dislocation (unstable)	IIA: presence of femoral head	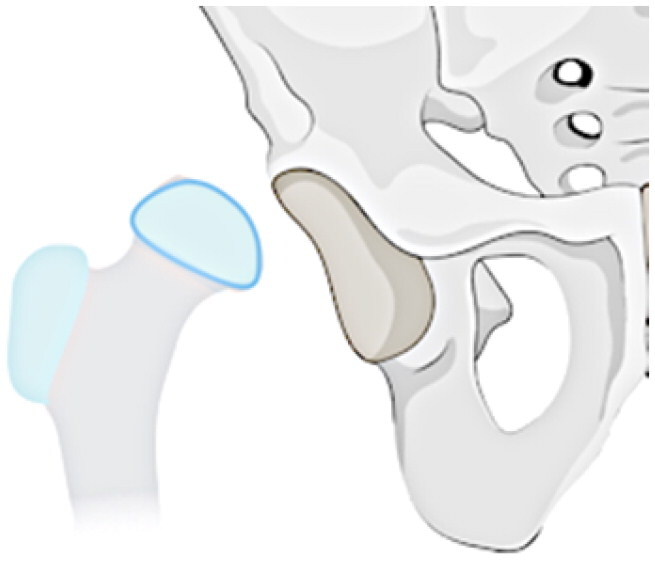
	IIB: femoral head absence	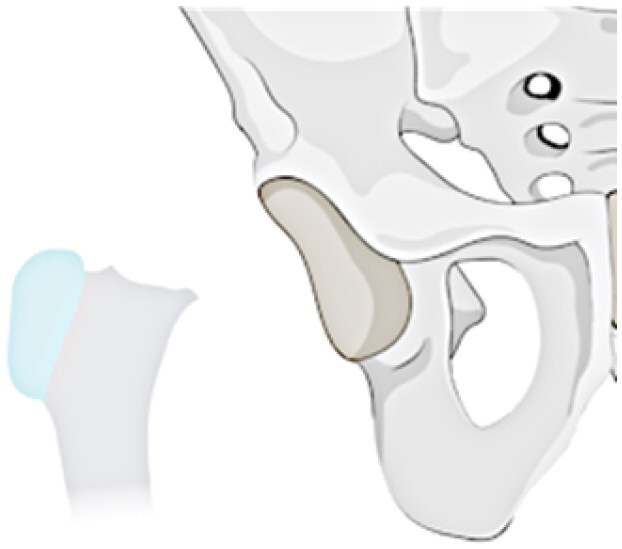

### Statistical analysis

All data were corrected for logical errors, encoded, and entered into the computer. We used IBM SPSS 26 statistical software(IBM, Armonk, NY) to analyze the data, with the significance level set at α < 0.05. To evaluate agreement, agreement rates and weighted kappa coefficients were calculated. The kappa coefficient ranges from −1 to 1, with the following interpretations: values ≥0.80 indicate perfect agreement; values between 0.61 and 0.80 represent substantial agreement; values between 0.41 and 0.60 suggest moderate agreement; values between 0.21 and 0.40 indicate fair agreement; and values ≤0.20 are considered slight or poor agreement.

## Results

Out of the 18 included hips, 10 males and 8 females;5 (27.8%) were classified as type IA, 6 (33.3%) as type IB, none as type IC, 2 (11.1%) as type ID, 3 (16.7%) as type IIA, and 2 (11.1%) as type IIB. Please refer to Table 2 for further details.

**Table 2.** Basic information and characteristics of patients.

Case	Hip	Age group at onset of infection (years	Pathogenic bacteria	Antibiotic	Drainage method	Other bones and joints involved	Sequelae classification(HHPO)	Reconstruction
1	R	10–12	MSSA	Vancomycin	AD	None	IB	None
2	L	10–12	MRSA	Vancomycin	AD	left distal tibia and left proximal humerus	ID	GTA+ FL
3	L	1–3	MSSA	Unknown	AD	None	IIA	Salter + PFO(varus)
4	L	1–3	Unknown	Unknown	AD	None	IIA	Pemberton + GTE + PFO
5	R	1–3	Unknown	Cefuroxime	PD	None	IA	None
6	R	13–15	MRSA	Vancomycin	AD	None	IIA	None
7	R	10–12	Unknown	Cefmetazole	AD	None	IB	None
8	R	1–3	MRSA	Vancomycin	AD	None	IA	None
9	L	1–3	*Citrobacter youngae*	Vancomycin	AD	right distal femoral	IB	None
10	L	10–12	Unknown	Cefuroxime	AD	None	IA	None
11	L	1–3	Unknown	Vancomycin	AD	right distal tibia	IIB	Chiari + PFO(varus)
12	L	10–12	Unknown	Vancomycin	AD	left ilium	IB	None
13	R	<1	MSSA	Vancomycin	None	None	ID	Salter + GTE + PFO(varus)
14	L	13–15	MRSA	Vancomycin	AD	None	IB	None
15	L	10–12	MSSA	Cephazolin	AD	None	IA	None
16	L	4–6	Unknown	Vancomycin	AD	None	IB	None
17	R	1–3	Unknown	Cephazolin	AD	None	IA	None
18	L	1–3	Unknown	Unknown	PD	None	IIB	PFO(varus)

*Note.* 1. FL, Femoral lengthening; 2. PFO(varus, Proximal femur varus osteotomy; 3. PFO(valgus, Proximal femur valgus osteotomy; 4. GTE, Greater Trochanteric Epiphysiodesis; 5. G TA, Greater trochanteric advancement; 6. AD, Arthrotomy drainage; 7. PD, Puncture drainage; 8. MSSA, Methicillin-sensitive *Staphylococcus aureus*; 9. MRSA, Methicillin-resistant *Staphylococcus aureus*.

## Risk factor analyses related to severity

### The age at the initial hip infection

Case 13 of the 18 cases reviewed had a septic infection of the right hip in the neonatal period and was the youngest patient in the acute phase of the inflammation with a sequelae of type ID; case 14 was the oldest patient with acute inflammation and was 13 years old; cases 3, 4 and 6 had a sequelae of type IIA; and cases 11 and 18 had a sequelae of type IAIIB. The study indicates that the severity of sequelae generally increases among those infected at a younger age. However, excluding patients without sequelae in this study prohibits determining whether the younger generation at the time of infection onset is associated with more severe sequelae.

### Multiresistant bacterium

In this study, MRSA was the known causative agent for six cases of hip infection, MSSA for four cases, and *Citrobacter youngae* for one case, while the causative agent remained unknown in the remaining seven cases. Of the patients infected with MRSA, three had sequelae of type IA, one had type ID, one had type IIA, and one had type IIB. It was impossible to ascertain whether there was a correlation between the causative organism’s resistance and the sequelae’s severity.

### Other factors

The current study found no correlation between the severity of sequelae and gender, laterality, drainage method during the acute phase or antibiotic category.

## Agreement analysis

### HHHPO interrater agreement and intrarater agreement

The results of the interrater agreement of the HHPO type are described in Table 3. The HHPO had moderate agreement for interrater agreements. The intrarater agreement analysis for eachobserver is described in Table 4. Each observer’s intrarater agreement varied from moderate to substantial.

**Table 3.** Interrater agreement results for the HHPO and Chio.

Type	Trial	percentage agreement(%)	Kappa score	95% CI
HHPO	1	64	0.52	0.42–0.62
	2	67	0.61	0.51–0.71
Chio	1	35	0.21	0.11–0.31
	2	46	0.32	0.22–0.42

95% CI, 95% confidence interval; HHPO, Honghui Pediatric Orthopaedic Classification; Chio, Choi classification.

**Table 4.** Each intrarater agreement results for HHPO and Chio.

Observer^a^	HHPO			Chio		
	percentage agreement(%)	Kappa score	95% CI	Percentage agreement(%)	Kappa score	95% CI
Chief physician^b^	83	0.71	0.58–0.84	52	0.35	0.16**–**0.55
Deputy chief physician^b^	78	0.69	0.49–0.81	55	0.38	0.17–0.57
Attending physician^b^	70	0.66	0.41–0.72	50	0.40	0.23–0.53
Attending physician^b^	73	0.60	0.45–0.76	53	0.41	0.24–0.55
Resident physician^b^	72	0.55	0.37–0.73	61	0.45	0.26–0.63
Resident physician^b^	68	0.59	0.41–0.70	53	0.41	0.25–0.55

95% CI, 95% confidence interval; HHPO, Honghui Pediatric Orthopaedic Classification; Chio, Choi classification.

^a^ The observer has listed a ranking in order of highest to lowest position; There are four stages of physician titles in the Chinese healthcare system: primary title (Resident Physician), middle title (Attending Physician), vice-senior title (Deputy Chief Physician), and senior title (Chief Physician). In general, each level of physician needs a 5-year interval of clinical accumulation to pass the qualification and examination before being promoted to the next level.

^b^ The rater is from the Honghui Hospital.

## Chio interrater agreement and intrarater agreement

The results of the interrater agreement of the Chio type are described in Table 3. The Chio type had substantial agreement for interrater agreements. The intrarater agreement analysis for each observer is described in Table 4. Each observer’s intrarater agreement varied from fair to moderate.

## Sequelae treatment

### Type I

**Figure 1.**
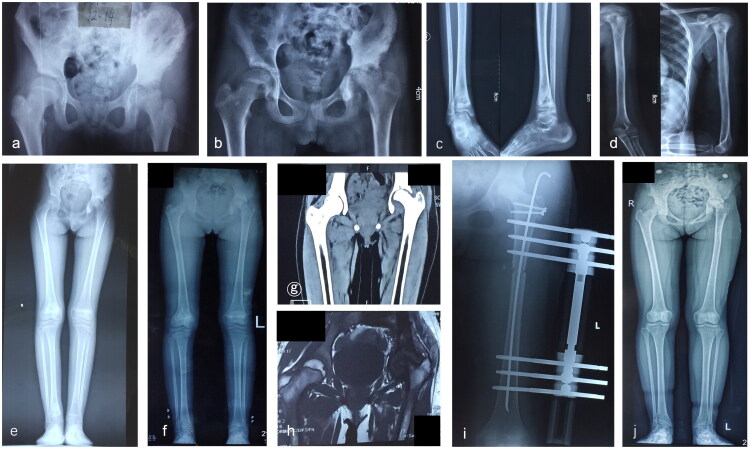
**a–j** The case 2, aged 10–12 years, experienced left-sided SAH secondary to a lung infection, and her inflammation was effectively controlled with intravenous vancomycin and oral linezolid while she underwent two hip arthrotomy drainages; however, during the acute period of illness, her focus of disease spread to multiple sites throughout her body (left distal tibia, left humerus, left ilium) (**a–d**); ultimately, one year later, the patient experienced ID sequelae in her left hip while also having LLD (**e–h**). She underwent a left GTA procedure combined with femoral osteotomy lengthening (**i** and **j**), resulting in satisfactory hip function and gait improvements.

**Figure 2.**
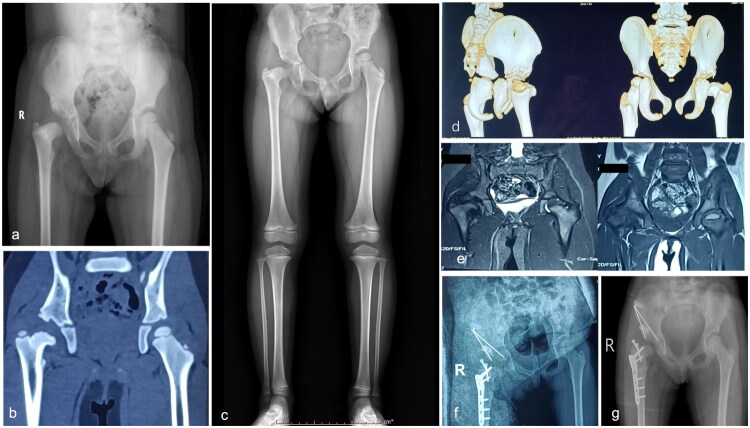
**a–g** The case 13, aged 4–6 years, with neonatal sepsis caused by right-sided SAH was discharged from the hospital with inflammation under control after intravenous vancomycin in an outside institution; the right hip was not incised and drained, and she was given oral cefmenoxime for one month. However, at four years of age, the patient experienced hip pain, dysfunction and gait abnormalities. The examination showed sequelae of the left hip (type ID) and LLD (16.4 mm) (**a–e**); finally, a salter osteotomy combined with GTE and proximal femoral de-rotation osteotomy was performed in our institution, and postoperatively, the right hip pain disappeared, and the function and gait were improved (**f** and **g**).

Of the cases observed in this study, 72.2% (*n* = 13) were classified as Type I. Notably, only two patients (15.4%) with Type ID underwent reconstructive procedures. Among these were Case 2 (as illustrated in Figure 1), who had an undislocated hip, complete femoral head resorption, 20 mm lower LLD, and hip pain and claudication gait. The patient underwent reconstruction by lengthening the femur through GTA. One year after surgery, the patient had regained limb length and exhibited good hip function and gait. In contrast, case13 (refer to Figure 2) exhibited right acetabular dysplasia (AI: 20°), resorption of the femoral head, a slight collodiaphyseal angle (105° on the right side, 140° on the left side), and a large femoral antegrade angle (34° on the right side, 17° on the left side), as well as LLD (16.4 mm), limited hip abduction (45° on the right, 50° on the left) and a claudication gait. Pelvic Salter osteotomy, greater trochanteric epiphysiodesis(GTE), and proximal femoral valgus osteotomy were performed. Reconstructive surgery was not undertaken for other Type I patients, as hip function and gait were less or less affected.

### Type II

**Figure 3.**
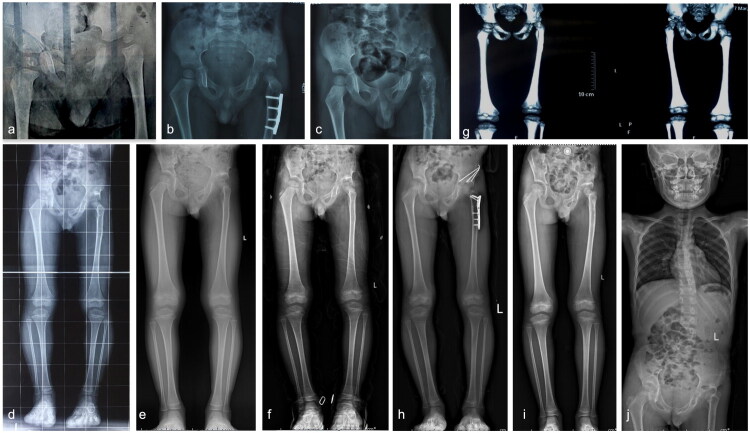
**a–j** The case 3, currently aged 7–8 years, developed left-sided SAH when he was less than 2 years old. He received intravenous antibiotics and incision and drainage treatment to control inflammation, which proved effective. However, he suffered sequelae of subluxation of the left hip (**a**) and underwent a proximal femoral ravus osteotomy (**b**) as a result. After the removal of the internal fixation device following lateral femoral osteotomy healing (**c**), close monitoring occurred until the child was seven years old (**d–g**). At that point, the child developed a claudication gait, which imaging confirmed as a significant right-sided femoral neck anteversion, inadequate hip correspondence, and lateral acetabular resorption. The patient underwent a combined proximal femoral de-rotation osteotomy with salrter osteotomy (**h**); postoperatively, the patient has a stable hip, limb shortening of <2 cm, compensatory spinal scoliosis, good hip function and an essentially normal gait, with removal of internal fixation devices after healing of the osteotomies (**i** and **j**).

**Figure 4.**
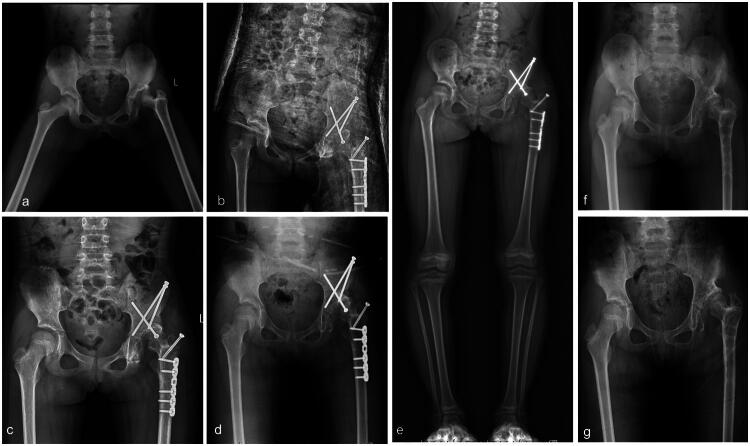
**a–g** The case 4, a child aged 7–8 years, developed left-sided SAH at 3–4 years of age. The inflammation was effectively controlled with antibiotics, hip arthroplasty, and drainage at other institutions. One year ago, she developed claudication and pain in the left hip. A pelvic X-ray showed a subluxation of the left hip with partial resorption of the femoral head (type IIA) (a). She underwent a pemberton osteotomy of the acetabular side combined with GTE and a proximal femoral osteotomy (**b**). Postoperatively, the osteotomy site healed, the hip joint was stable, the acetabulum was satisfactorily accommodated, there were no residual complications such as LLD, and the hip joint functioned well more than one year after surgery (**c–e**)—removal of internal fixation devices (**f** and **g**).

**Figure 5.**
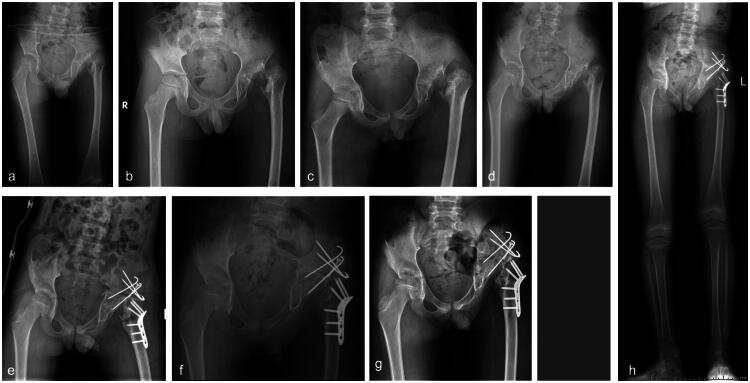
**a–h** The case 11, a child aged 9–10 years, developed left-sided SAH at less than 2 years of age, which caused a gait abnormality noticed when he was five years old. A pelvic X-ray revealed left hip dislocation with complete femoral neck resorption and residual femoral head (type IIB) (a). No improvement was observed during consecutive follow-ups (b–d), and he developed left hip pain. As a result, he underwent acetabular lateral Chiari osteotomy combined with proximal femoral valgus osteotomy (e–g). Recent follow-up showed osteotomy healing, hip stability and good hip function, but a residual LLD of 3 cm remains (h).

**Figure 6.**
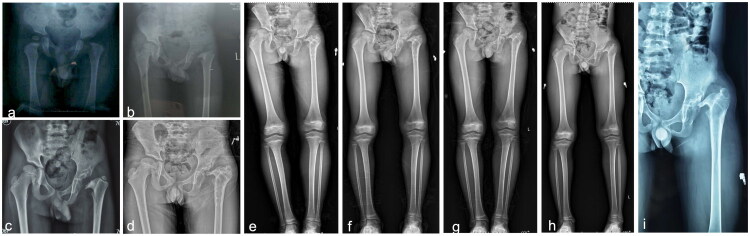
**a–i** The case 11, a child aged 12–13 years, developed left-sided SAH at 1-2 years of age, resulting in residual left hip dislocation, acetabular dysplasia, and deformity with loss of the neck of most of the femoral head (**a**). He underwent proximal femoral exostosis osteotomy to improve his gluteus medius gait, lengthen his limb and stabilise his hip (**b–d**). However, during subsequent follow-up appointments following the removal of the internal fixation device, it was discovered that the left hip remained dislocated. Specifically, after the device was removed, the left hip was found to be dislocated with a difference in leg length of less than 2 cm (**e–i**). As a solution, it was decided to perform an acetabular osteotomy in combination with GTA to reconstruct the femoral head and stabilise the hip joint. However, after the affected limb was fitted with appropriate shoe lifts,the patient’s left hip function is currently acceptable, with Nearly painless and impact on daily life. Additionally,the parents lack confidence in reconstructive surgery, and therefore, the surgical treatment has not been pursued. The patient is still undergoing follow-up.

In five patients (27.8%) with type II, three patients with type II underwent reconstructive surgery. Of these, two were of type IIA (case 3 and case 4). In case 3 (refer to Figure 3), Salter osteotomy and proximal femoral de-rotational osteotomy were performed; in case 4 (refer to Figure 4), Pemberton osteotomy of the pelvis, GTE and proximal femoral osteotomy were performed. Post-surgery, there was a significant enhancement in hip functionality and gait. Another patient with type IIB (case 11, see Figure 5) underwent pelvic Chiari osteotomy and proximal femoral osteotomy, significantly improving hip function and gait post-surgery. A second patient (case 18, see Figure 6) underwent proximal femoral valgus osteotomy and femoral lengthening 5 years after sequelae onset (type IIB) to address gait abnormalities and enhance gluteus medius gait while maintaining hip stability. However, after removing the internal fixation device, the deformity continued, although the affected hip did not experience significant pain and functioned adequately. Reconstructive surgery was not conducted presently due to the parent’s lack of confidence in undergoing reoperation.

## Discussion

SAH is a joint infection caused by bacteria that can occur in isolation or due to osteomyelitis spreading to nearby joints [18]. Its incidence is about 5/10,000 to 12/10,000, with a similar prevalence in both sexes [19]. And it generally occurs in younger age groups, such as newborns, infants and toddlers aged 2–3 years. Among other things, the diagnosis of neonatal SAH presents some difficulties [20–22]. After confirmation of the diagnosis, the use of antibiotics and emergency arthrotomy drainage and lavage is the mainstay of treatment [23], which can reduce the risk of complications and achieve a good prognosis after timely and effective treatment [24]. Repeat Hip Aspiration (RHA) [23]and arthroscopic surgery [25] have also been performed, all with good short-term prognostic outcomes. Unfortunately, delays in diagnosis and treatment can have devastating consequences for the child, resulting in a reduced quality of life, frequent medical treatment, and increased economic and social burden.

Several factors associated with the development of sequelae have been reported in previous studies, including age at the time of infection, prematurity, intensive care, bacterial genus and delay in treatment [26–28]. In addition, delayed diagnosis of SAH beyond four days has also been suggested to increase the risk of poor prognosis [29]. In the present study, there was a tendency for the younger the age of possible acute infection, the more severe the sequelae. We hypothesise that the anatomy of the femoral neck and head may be related to the poor prognosis of younger patients. Firstly, the intra-articular location of the femoral neck epiphysis, combined with the presence of trans-epiphyseal plate blood vessels, creates an environment conducive to the transmission of pathogenic bacteria through the bony structures and into the joints [30,31]; secondly, in the period when the primary blood supply to the femoral head is from the epiphyseal artery [32], heightened joint cavity pressure could result in thrombosis of the epiphyseal artery, leading to ischaemic necrosis of the femoral head [33,34].

Reasonable and correct classification of sequelae is essential to guide treatment and evaluate prognosis. It has been clinically observed that confident children who have experienced SAH may exhibit sequelae of varying severity once the inflammation has been controlled. Such deformities can range from mild alterations of the femoral head, structural disturbances of the femoral head and neck, complete destruction and absence of the femoral head, and hip dislocation. The classification method proposed by Choi et al. describes the type and severity of sequelae after SAH and is the most widely accepted classification method. However, the classification method includes too many classes and subtypes – four significant types and eight subtypes – and needs to be more complex and detailed to facilitate a more reasonable treatment plan and prognosis. Furthermore, it is essential to note that this classification method exhibits low inter-observer consistency. For example, Forlin and Milani et al.’s [16]study found that only 7 out of 41 cases were entirely consistent with the classification outcomes, and a similar result was found in the present study.

Assessing the classification of young children is a more challenging issue because their skeleton is mainly cartilage, and the hip joint is not fully visible on radiographs until the skeleton has matured [35,36]. Cartilage structures, precisely, must be visualised via arthrography or joint MRI. Nonetheless, these exams frequently necessitate sedation or anaesthesia, especially in young children. All in all, radiographs continue to be the favoured approach for classification. Ultimately, the proposed study advances a modified classification scheme for anteroposterior hip radiographs based on two key factors: the severity of femoral neck and head structural disturbances and the stability of the hip, following the principles of simplicity, comprehensibility and memorability, and regarding the Choi et al. classification scheme. Refer to Table 1 for a schematic diagram.

The results of the present study showed that the HHPO classification performed better in agreement than the Choi classification. Furthermore, our observations indicate that the prognosis is poorer the more posterior the HHPO classification is, which is consistent with what we have observed in clinical practice. In conclusion, the HHPO classification comprises only two primary types that are more concise and easier to comprehend than the previous version. Additionally, the foundation of the classification is clearly defined while taking into account varying severity of femoral head and neck structural damage, deformity, and joint stability, which is in line with the principle of practicality and easy to memorise or better guide clinical practice. However, there is no clear reference standard for the timing and modality of all reconstructive surgery in this study, which is mainly based on the assessment of the children’s clinical symptoms and hip imaging by the attending surgeons and the implementation of their respective modalities, which they are relatively good at. Furthermore, our proposed HHPO typing was not used preoperatively in these patients, so its practical value needs to be further verified. In addition, the timing of the diagnosis of SAH residual deformity and the timing of reconstructive surgery varied widely among the 18 patients included in this study, and premature staging of residual deformity when the progression of residual deformity has not been stabilised and self-contouring has not been completed may lead to overtreatment. Therefore, it is necessary in the future to incorporate the time factor into staging to more accurately assess residual deformity and thus develop more rational treatment plans.

Reconstructive or salvage surgery is designed to provide optimal biomechanical support to the hip and lower limb at skeletal maturity to reduce the risk of early retrogression arthritis [37,38] By restoring the stability of the hip, correcting the deformities of the proximal femur and acetabulum, improving the match of the hip, preserving the mobility of the hip, eliminating potential causes of pain and, finally, balancing the pelvic level and the length of the lower limb without residual axial or rotational deformities.

Children with type I may be asymptomatic in early childhood, but clinical symptoms such as pain, claudication, joint instability or contractures, limited mobility, LLD, and secondary scoliosis do not appear until adolescence. Therefore, early treatment is more critical for this group of children. Treatment aims to correct pathological alterations such as abnormal femoral neck stem angle, inadequate acetabular coverage, short neck deformity and pseudoarthrosis. Given the better stability of this type of hip, the overall prognosis is more satisfactory if a reasonable, individualised treatment plan is made available.

Type II is a more severe sequelae, and treatment focuses on achieving hip reduction and maintaining hip stability. Type IIA can be performed by pelvic osteotomy combined with proximal femoral osteotomy for joint repositioning and stabilisation, e.g. pelvic triple osteotomy, Ganz osteotomy combined with proximal femoral varus osteotomy. In children with type IIB, replacement of the absent femoral head by reversal of the greater trochanter in combination with a pelvic osteotomy has been studied to achieve hip reduction and stabilisation [39,40]. Ilizarov developed and popularised a new procedure, the Ilizarov Hip Reconstruction (IHR), which, in addition to performing a proximal femoral varus osteotomy, corrects the varus deformity of the knee by performing a distal femoral varus osteotomy and femoral lengthening to correct LLD [41–44] In addition, previous studies [45,46]have reported medium and long-term follow-up results with this procedure, showing good limb balance and hip function. However, this procedure is relatively complex and requires attention to various intraoperative details to achieve surgical success. Recent studies demonstrate promising outcomes with single-stage THA for adult septic hip sequelae, including cases with active infection [47–50].However, pediatric applications are limited by skeletal immaturity. Research to further improve implant survival as well as the ease of revisions in teenagers is needed.

Our data provide a preliminary framework requiring validation in larger, multi-center studies with extended follow-up to confirm classification reliability and treatment efficacy. The HHPO classification may be more applicable to cases requiring reconstruction than to the full spectrum of SAH sequelae.

The limitations of this study are as follows:

1. The sample size is relatively small, which affects the accuracy of the imaging study results. More samples should have been included, but the incidence of SAH (Subarachnoid Hemorrhage) itself is low, and even fewer patients visit our medical institution. Currently, we can only include these relatively complete patient data. The cohort size (*n* = 18) limits statistical power and warrants validation in larger cohorts.
2. There are no complete records and reports on functional rehabilitation, and the assessment of surgical efficacy is limited to imaging examinations. The main reason is that rehabilitation medicine is underdeveloped in our medical institution. Postoperative rehabilitation for patients is conducted by parents following the outpatient doctor’s verbal instructions, resulting in a significant variation in early outcomes. However, possibly due to the age factor of the children, most of them eventually achieved relatively satisfactory functional recovery. Unfortunately, there are no complete medical records for this part. Insufficient long-term data for non-operative subgroups limits assessment of treatment durability.
3. This study is retrospective, and the modified classification was not applied before the reconstruction surgery in these cases. The idea of modifying the classification arose when we reviewed these cases. We hope to apply this classification in future cases to understand its true practical value.

## Conclusion

Based on our preliminary investigation, we found that the HHPO classification exhibited superior consistency. It is straightforward, comprehensible, and memorable, offering advantages for physicians in treatment planning and prognostic assessment. There appears to be a propensity for more severe sequelae when the infection occurs at a younger age. This classification system provides the optimal solution. The HHPO classification demonstrates potential clinical utility in this small cohort study.

## Data Availability

Data are available for review. If anyone wants to request data from this study, please get in touch with the first author, Bohai Qi.
